# Comprehensive understanding of anchorage-independent survival and its implication in cancer metastasis

**DOI:** 10.1038/s41419-021-03890-7

**Published:** 2021-06-18

**Authors:** Zhong Deng, Huixue Wang, Jinlong Liu, Yuan Deng, Nu Zhang

**Affiliations:** 1 Department of Neurosurgery, The First Affiliated Hospital of Sun Yat-sen University, Guangzhou, P. R. China; 2 Institute of Precision Medicine, The First Affiliated Hospital of Sun Yat-sen University, Guangzhou, P. R. China; 3 Department of Ophthalmology, Shanghai Ninth People’s Hospital, Shanghai Jiao Tong University School of Medicine, Shanghai, P. R. China; 4 Shanghai Key Laboratory of Orbital Diseases and Ocular Oncology, Shanghai, P. R. China

**Keywords:** Metastasis, Cell death

## Abstract

Detachment is the initial and critical step for cancer metastasis. Only the cells that survive from detachment can develop metastases. Following the disruption of cell–extracellular matrix (ECM) interactions, cells are exposed to a totally different chemical and mechanical environment. During which, cells inevitably suffer from multiple stresses, including loss of growth stimuli from ECM, altered mechanical force, cytoskeletal reorganization, reduced nutrient uptake, and increased reactive oxygen species generation. Here we review the impact of these stresses on the anchorage-independent survival and the underlying molecular signaling pathways. Furthermore, its implications in cancer metastasis and treatment are also discussed.

## Facts

1. There are four different forms of anchorage-independent survival, such as anoikis, autophagy, entosis, and cell cycle arrest, reported in the literature.
2. After detaching from the extracellular matrix (ECM), both the nonmalignant and malignant cells will be exposed to multiple stresses, including loss of growth stimuli from ECM, altered mechanical force, cytoskeletal reorganization, reduced nutrient uptake, and increased reactive oxygen species (ROS) generation.
3. Multiple signaling pathways, including integrin transduction and its downstream signaling pathways, such as paxillin/p130CAS, Ras-ERK, PI3K/AKT, Rho/ROCK, and YAP/TAZ pathway, are activated during detachment and contribute to anchorage-independent survival.
4. As the initial step of cancer metastasis, anchorage-independent survival shares many similarities with cancer metastasis, especially in regulation and activation of integrin transduction and its downstream signaling pathways.
5. Blocking integrin transduction and its downstream signaling pathways suppresses cancer metastasis; however, there emerges clinical treatment resistance.

## Open questions

1. Is there any other form of anchorage-independent survival for detached cells? What is the regulation and mechanism in cancer metastasis?
2. How to establish in vitro models to mimic anchorage-independent survival, in vivo, and to study the regulation and mechanism of anchorage-independent survival?
3. How can we overcome treatment resistance of targeting therapy in cancer metastasis?

## Introduction

Attachment, which mainly depends on cell–ECM interactions, is one of the most important factors regulating cellular morphology, dynamic, behavior, and finally, cell fate in both normal and malignant cells. In normal cells and tissues, there is a dynamic balance between attachment and detachment in maintaining cell survival and homeostasis [1], and disruption of this balance could contribute to malignant transformation [2]. In malignant cells and tissues, both attachment and detachment contribute to cancer progress. Attachment promotes the growth of cancer cells; however, detachment initiates cancer metastasis, which causes 90% of human cancer deaths [3]. Once detached from the ECM, both the normal and transformed cells present a round cell shape and are exposed to a completely different chemical and mechanical environment. In turn, the environmental chemical and mechanical stresses will challenge the cell fate. Regarding that, we first review different forms of anchorage-independent survival reported in the literature. Then we discuss the impact of environmental stresses on anchorage-independent survival during detachment. The underlying molecular signaling pathways involved in anchorage-independent survival are also reviewed. Given their critical role in metastasis, we finally discuss the implications of anchorage-independent survival in cancer metastasis and treatment.

## Different forms of anchorage-independent survival

**Fig. 1:**
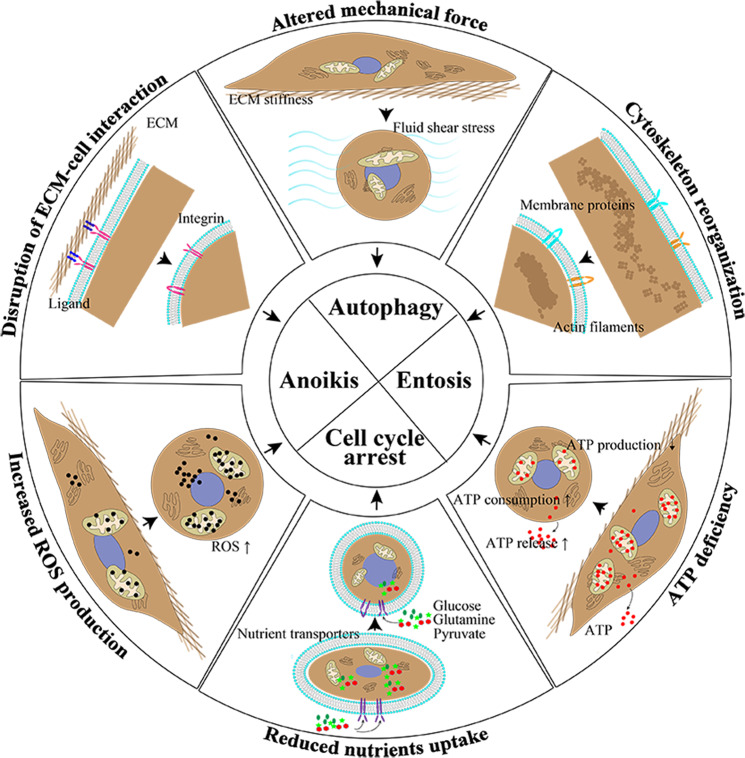
The anchorage-independent survival and cellular and environmental changes during detachment. After losing anchorage to ECM, the cells are exposed to a totally different environment and present multiple cellular changes, including disruption of extracellular matrix (ECM)–cell interaction, altered mechanical force, cytoskeleton reorganization, ATP deficiency, reduced nutrient uptake, and increased reactive oxygen species (ROS) production. **A** Disruption of extracellular matrix (ECM)–cell interaction. Integrins lose the binding of ligands and stimulation from the ECM. **B** Altered mechanical force. The main mechanical force shifts from ECM stiffness to fluid shear stress. **C** Cytoskeleton reorganization. The cells present a round cell morphology and the membrane proteins undergo structure deformation and activation. **D** ATP deficiency. ATP deficiency is a result of reduced ATP production, increased ATP release, and enhanced ATP consumption. **E** Reduced nutrient uptake. The uptake of glucose, glutamine, and pyruvate is reduced in detached cells due to various reasons. **F** Increased ROS production. The ROS generation is increased during detachment. As a result, the detached cells undergo anoikis, autophagy, entosis, and cell cycle arrest.

The correlation of cell adherence and growth was first unveiled by *MacPherson* and *Montagnier* in 1964 [4]. In 1975, *Folkman* and *Greenspan* demonstrated the importance of anchorage for cell growth control [5]. The following researchers proved the importance of anchorage-independent survival in normal tissue dynamics and cancer progress. Four different forms of anchorage-independent survival, including apoptotic cell death (anoikis), nonapoptotic cell death (including autophagy and entosis), and cell cycle arrest, are reported in the literature (Fig. 1).

### Apoptotic cell death: anoikis

In 1993, *Meredith* and colleagues found that loss of attachment to ECM could induce cell death in several cell types [6]. Once detached, the endothelial cells and gut epithelial cells presented the typical apoptotic morphology indicated by cell morphological changes and nuclear fragmentation; however, ureteral epithelial cells exhibited a distinct morphology [6]. The apoptotic phenotype in suspended epithelial cells was also observed by *Frisch* and *Francis*, and they termed this phenomenon “anoikis”–the ancient Greek word for “homelessness” [7]. The following studies proved its critical role in the homeostasis of skin [8], digestive tract [9], and mammary gland [10], as well as in physiological processes, such as fibrinolysis [11], aortic valves [12, 13], and vascular remodeling [14]. Intrinsic cell death molecules such as Bcl-2 family molecules and cytochrome *c*, extrinsic cell death molecules such as TNFR, DR5, or Fas, and other signaling molecules such as integrins and EGF reportedly modulate anoikis, see reviews for further information [15, 16].

### Nonapoptotic cell death

The results that ureteral epithelial cells exhibited a nonapoptotic morphology from *Meredith*’s study [6] and that blocking proapoptotic signaling pathway conferred partial but not complete resistance to anoikis [7] imply the presence of nonapoptotic cell death in detached cells.

### Autophagy

Autophagy is an adaptive response to a variety of integrated cellular and microenvironmental stresses, such as deprivation of nutrients, oxygen, and growth factor [17, 18]. The presence of autophagy in detached normal human mammary epithelial cells (hMECs) was proved by the observation of cytoplasmic vacuoles in the dying central cells in 3D suspension culture in vitro [1]. Induction of autophagy promoted cell survival in detached nontransformed epithelial cells and primary fibroblasts [19, 20]. Researchers also identified the protective role of autophagy in suspension or spheroid-cultured malignant cells, such as breast cancer, fibrosarcoma, glioma, ovarian cancer, and lung cancer [21–25]. Molecules and signaling pathways regulating redox metabolism and cell growth are critical regulators in autophagy during detachment, so are those involved in cell detachment and cytoskeleton organization, such as ERK/AMPK, mTOR, integrins, and GTPase [17, 26, 27].

### Entosis

Entosis is a process involving cell engulfment first observed in detached cells [28]. *Overholtzer* and colleagues documented cell-in-cell structure and termed as “entosis” in several suspension-cultured nontumorigenic cells and tumor cells. Cell internalization increased with the elongation of cell detachment independent of apoptosis. Moreover, the internalized cells might undergo cell death by lysosomal digestion, division, or release [28]. Once documented, entosis was found in multiple malignant disease, including breast cancer, colon carcinoma, stomach carcinoma, cervical carcinoma, liver carcinoma, melanoma, lung small cell carcinoma, prostate cancer, and pancreatic cancer, in vivo and in vitro [28–34]. Interestingly, the cell-in-cell structure is much more common in fluid-derived cancer samples, in vivo [35–37]. By now, E-cadherin, α-catenin, and RhoA GTPase are necessary and sufficient to induce the formation of cell-in-cell structure, and autophagy pathway proteins are required for entotic cell death [38]. In addition, recent report finds that genetic features are significantly associated with entosis, such as TP53 mutation, KRAS amplification, and c-myc amplification [38, 39].

### Cell cycle arrest

Cell cycle arrest with the cease of cell growth and DNA synthesis was also observed in normal and transformed epithelial cells during detachment, which could be reversed by cell reattachment [40]. Similarly, fibroblast cells underwent reversible cell growth withdrawal and arrest of mRNA production and protein synthesis when exposed to suspension condition [41, 42]. Further studies confirmed its presence in normal and transformed epithelial and endothelial cells, fibroblasts, and smooth muscle cells under suspension condition, and it is noteworthy that cells are arrested in G1 phase during detachment [7, 43–46]. It is also worth mentioning that some groups propose cell cycle arrest as one of the mechanisms to acquire anoikis resistance [44, 46, 47]. These studies proved that integrins and cell cycle inhibitors, such as p27 and p57, could induce cell cycle arrest in suspended cells [43–46, 48].

## The environmental and cellular stresses during detachment

### Disruption of cell–ECM interactions

Cell–ECM interactions mainly depend on the architecture of focal adhesions (FAs) and cytoskeletal proteins. FAs are integrin-based multiprotein complexes composed of ~160 distinct components including activation and inhibition molecules of integrins, signaling molecules (kinase, phosphatases, and G proteins and their regulators), and actin filaments. Cytoskeletal proteins are actin-based structures and regulate cell shape and motility by changing cytoskeleton organization. Physically, FAs interconnect with cytoskeletal proteins via the ends of actin filaments. Functionally, there are feedback networks between cytoskeleton reorganization and integrin activation. For further information, see reviews [49, 50] and Box 1.

It is well established that integrins protect cells against anoikis [15, 16], and worthy to note that the protecting role of different integrins differs in different cell types. For example, integrin α_V_β_3_ is required for angiogenic vascular cell survival during detachment [51]. However, it is dispensable for the survival of suspended MG-63 human osteosarcoma cells [52] and melanoma cells [53]. Moreover, acinar morphogenesis of human breast epithelial cells could be blocked by anti-β1 or anti-α2 integrin antibody but not by anti-α3 integrin antibody [54]. These studies showed that the expression, translation, degradation, and function of integrins in different cell types might account for these differences [54, 55].

As mentioned above, autophagy is increased during detachment and protects cells against stresses from ECM detachment [17, 26]. In suspension conditions, the function of integrin is downregulated because of lacking ligand binding; hence, integrin inhibition by a specific antibody or cilengitide, an αv integrin antagonist, could induce autophagy [56, 57].

Similarly, disregulation of integrin signaling induces cell cycle arrest of suspended cells [44, 47, 58–60]. Deletion of β4 integrin cytoplasmic domain leads to epithelial cells detachment and cell cycle defects, in vivo [60]. Vice versa, overexpression or activation of downstream kinases of integrin pathway, such as protein kinase C, ERK, and ILK, protects cells from cell cycle withdrawal [47, 58, 59].

### Altered mechanical forces and cytoskeleton reorganization

Besides reciprocal relations between integrin and cytoskeletal organization, mechanical force is another major factor regulating cytoskeleton dynamics and cell survival [61]. The major source of mechanical force for the attached cells comes from the biophysical property of ECM (e.g. stiffness) and interstitial fluid pressure; however, the main mechanical stress for the detached cells derives from fluid-based mechanics, such as fluid shear flow [62]. Environmental mechanical force induces biochemical signaling cascades through modulating the activation of mechanosensitive proteins and cytoskeletal dynamics, which were termed as “mechanical transduction”. Please refer the reviews for further information [61, 63, 64]. Additionally, mechanical force and cytoskeleton reorganization could directly modulate the protein activity of integrin and its adaptor proteins by regulating their 3D structure and binding affinity [61, 65, 66]. For example, force applied to Notch-ligand bond could also expose a cleavage site of Notch to initiate Notch and integrin signaling activation [66]. Cell membrane deformation induces opening and activation of mechanosensitive PANX1 channels, which permits cell recovery from traumatic deformation [67].

A body of studies have proven that increased mechanical force, either from contracted ECM or increased fluid shear flow, in vitro and in vivo, promotes apoptosis [68], autophagy flux [69], and G1–S cell cycle transition [70, 71]. Despite that the cells in these studies are cultured in attached conditions, it is possible that altered mechanical force and cytoskeleton organization could also modulate anoikis, autophagy, entosis, and cell cycle in a similar manner under detached conditions. Further efforts are needed to establish effective models to investigate the impact of altered fluid shear stress on cell survival in suspension culture.

### ATP deficiency, reduced nutrient uptake, and increased reactive oxygen species generation

ATP deficiency, reduced nutrient uptake, and increased reactive oxygen species (ROS) generation are ubiquitous in cells deprived of ECM. ATP deficiency usually results from enhanced ATP releasing, increased ATP consumption, or reduced ATP production. Increased ATP releasing is found in cells activated by shear stress, which could augment mitochondrial ATP generation [72]. Membrane deformation also induces increased ATP releasing, and the released ATP in turn suppresses deformation-induced apoptosis of vascular metastatic breast cancer cells [67]. Under the altered mechanical force, ATP consumption is also enhanced to maintain the dynamics of actin cytoskeleton [73].

Remarkably, ATP production is diminished as the result of decreased nutrient uptake during detachment [74]. Enhanced glucose uptake by ERBB2 overexpression restores ATP production and facilitates cell survival [74]. Restricted uptake of three key carbon sources (glucose, glutamine, and pyruvate) during detachment is also recorded [48]. Thus, the flux through glycolysis, the pentose phosphate pathway, and the TCA cycle is reduced, all of which could be reversed by downregulation of PDK4, an important PDH inhibitor. In addition, the authors find that PDK4 expression is increased in detached cells and correlates with the expression of cell cycle inhibitors (p27 and p57) in 3D suspension culture system, which results in cell growth arrest [48]. The enhanced activity of PDK4 is also found in detached hMECs, and depletion of PDK4 increases glucose oxidation and ROS production, and hence results in heightened anoikis. However, PDK4 is overexpressed in human cancer cells and contributes to anoikis resistance [75]. Similar trends for glutamine metabolism that increased glutaminolytic enzyme GDH1 expression promote ATP production and anoikis resistance has been unveiled as well in detached lung cancer cells [76].

Until now, the precise reasons for reduced nutrient uptake during detachment are largely unknown. Glucose transporter (GLUT) 1 and 4, two members of the major glucose-uptake protein family, are generally stored in cytoplasmatic vesicles and can be transported to the plasma membrane along the actin cytoskeleton [77, 78]. Disrupting actin cytoskeleton assembly results in reduced glucose uptake [78]. Moreover, cytoskeleton reorganization might also impair the activity of nutrient-uptake channels or receptors on the membrane [61, 65]. These results indicate that cytoskeleton reorganization may account for nutrient starvation during detachment.

Despite the above elegant studies, the precise manner in which ROS is modulated during ECM detachment remains incompletely understood. However, the antioxidant defense is enhanced to promote anchorage-independent survival and inhibition of antioxidant defense causes cell death during detachment. For example, the expression of antioxidant enzymes, including catalase and superoxide dismutase (SOD2), is upregulated in detached hMECs, and both antioxidant compounds and overexpression of these antioxidant enzymes decrease the ROS level, enhance ATP generation and promote survival of detached hMECs [79]. What is more, silencing antioxidant gene expression in breast cancer cells results in compromised ATP production and limited anchorage-independent growth [79]. Endoplasmic reticulum (ER) stress signaling pathway is also activated under suspension, and inhibition of PERK and eIF2α, two important regulators in ER stress signaling pathway, decreases anchorage-independent cell survival [21, 80, 81]. More specifically, ATF4, another master transcription factor of ER stress signaling, activates the coordinated program of cytoprotective autophagy and antioxidant response through upregulation of the major antioxidant enzyme heme oxygenase 1 (HO-1), and hence protects detached colorectal fibrosarcoma cells from anoikis and promotes their lung colonization in nude mice [22].

## Important signaling pathways contributing to anchorage-independent survival

### Integrin transduction and its downstream signaling pathway

**Fig. 2:**
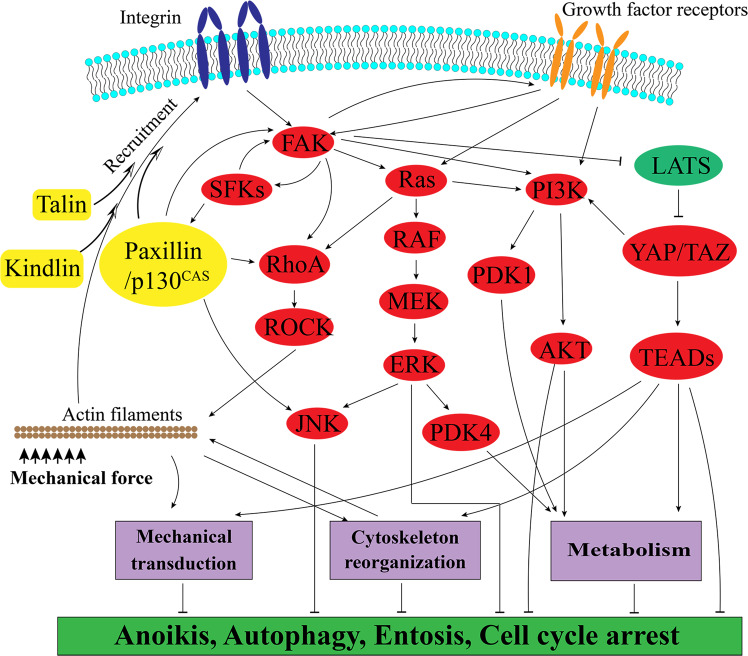
Molecular pathways sustaining anchorage-independent survival. During detachment, the ligand–integrin interaction between cells and ECM is disrupted and the cells lose the growth stimuli from ECM. However, the integrin can be also stimulated by mechanical force and cytoskeleton reorganization that actin filaments recruit integrin adaptor proteins, such as talin, kindlin, paxillin, and p130^CAS^ to integrin, hence inducing integrin clustering and activation. The activated integrin induces FAK/SFK activation and its downstream signaling proteins. There is also crosstalk between FAK/SFK and growth receptor signaling, such as EGFR, PDGFR, VEGFR, and IGFR signaling pathway. In cooperation with growth receptor signaling, integrin/FAK/SFKs induces Paxillin/p130^CAS^/JNK, Ras/ERK, PI3K/AKT, YAP/TAZ, and RhoA/ROCK signaling activation, and hence regulate mechanical transduction, cytoskeleton reorganization, and metabolism. Noteworthy, there is also crosstalk between these downstream signaling pathways. Eventually, activated integrin signaling and its downstream signaling inhibits various forms of cell death, including anoikis, autophagy, cell cycle arrest and entosis.

Integrin signaling is the major signaling pathway connecting the extracellular and intracellular environment. After losing anchorage to ECM, the environmental factors and intracellular changes, as reviewed above (Fig. 1), directly or indirectly modulate the activation of integrins and their downstream signaling pathways (Fig. 2). The main downstream molecules of integrins are FAK and SFKs. Worthy mentioning, only specific integrins (β1, β3, β5, and α11) were able to stimulate FAK/SFK phosphorylation [82, 83]. Additionally, there are cooperative interactions between integrin/FAK/SFKs pathway and growth receptor pathways. With or without cooperation with growth receptor pathways, FAK/SFK provoke downstream signaling pathways, including paxillin/p130^CAS^, Ras-ERK, PI3K/AKT, Rho/ROCK, and YAP/TAZ pathways [64, 84, 85].

### FAK/SFK-paxillin/ p130^CAS^ bidirectional signaling pathway

Activated by integrins, FAK/SFKs recruit and activate integrin adaptor proteins (IAPs), including talin, kindling, paxillin, and p130^CAS^ (also known as BCAR1) [63, 64, 86, 87]. On the other hand, mechanical force induces conformational changes of actin cytoskeleton and hence triggers recruitment and activation of those IAPs, which in turn phosphorylate and activate FAK and integrin [63, 86, 87]. Overall, it is a bidirectional pathway between FAK/SFKs and paxillin/p130^CAS^ regulating cell survival. As reported, FAK overexpression rescues detached hMECs and fibroblast cells from anoikis [88], while FAK inhibition reverses anoikis resistance and blocks protective autophagy in multiple cancers [89, 90]. The similar role of SFKs in anchorage-independent survival has been identified as well [89–91]. Consistently, paxillin and p130^CAS^ are upregulated in detached cancer cells and involved in FAK/SFK induced cell survival during detachment [92–94]. Notably, it is demonstrated that paxillin is essentially required for facilitating anchorage-independent survival via phosphorylating FAK [95].

### Cooperative growth receptor and death receptor signaling

During detachment, integrin/FAK/SFK pathway regulates the expression and activity of growth factor receptors, including EGFR, PDGFR, VEGFR, HGFR, and IGFR, see review [96]. It is also reported that activated growth factor receptor signaling pathways involve in integrin activation and recycling [97–99]. Hence, integrin/ FAK/SFKs and growth receptor pathways are closely cooperated to ensure anchorage-independent survival [24, 96, 100, 101].

On the other hand, there is a crosstalk between integrin/FAK/SFKs and death receptor pathways. For example, receptor-interacting protein (RIP) acts as a key shuttling protein between integrin/FAK signals and Fas/FasL signals. After dissociating from FAK, RIP binds to Fas and forms a death-inducing signaling complex, which activates caspase-3 and eventually results in anoikis [102]. In addition, death receptors such as FasL, DR5, and TNFR, are downregulated or inactivated in suspended cells against anoikis [102, 103].

### Ras/ERK signaling pathway

Early study reveals that Ras overexpression induces malignant transformation and protects epithelial cells from anoikis [7]. Ras/ERK upregulation and the de novo Kras mutation are detected in anoikis-resistant endothelial cells and cancer cells [2, 104]. Overexpression of key molecules in Ras/ERK signaling pathway attenuates cellular stress and promotes anchorage-independent survival [48], while Ras/ERK pathway inhibitors reverse anoikis resistance [2, 105]. Moreover, ERK activation, independent of serum and FAK or PAK activity, during detachment is sustained longer than growth factors induced activation [106]. These results indicate the protective and essential role of Ras/ERK signaling in response to detachment Box 1.

Box 1 Integrin activation and its role in cell survival The integrins comprise a family of 24 different heterodimers and they are assembled by 18 α and 8 β subunits with distinct ligand-binding specificities and signaling properties [84, 85]. Proteins from ECM, such as laminin, fibronectin, vitronectin, and collagen, are the major ligands of integrins [84]. Ligand-binding triggers integrin activation and initiates integrin-binding adaptor proteins binding to integrin cytoplasmic domain, and hence leads to actin cytoskeleton reorganization, integrin clustering, and fully activation. Consequently, fully activated integrin induces activation of focal adhesion kinase (FAK) and SRC family kinases (SFKs) and their downstream signaling pathways, which in turn control survival, proliferation, autophagy, and other cell fate transitions [64, 84, 85]. Thus, detachment will challenge cellular dynamics, behavior, and cell fate via modulating integrin signaling pathway.

### PI3K/AKT signaling pathway

Similarly, PI3K/AKT signaling pathway can be activated by detachment and protects cells from death during detachment [74, 105]. Previous studies demonstrate that PI3K/AKT downstream proapoptotic and antiapoptotic molecules, including Bcl-2, Bak, Bcl-X(L), and Bax, modulate anoikis in transformed and nontransformed cells [7, 15, 16]. The following studies prove that PI3K/AKT signaling pathway, cooperating with or without Ras/ERK signaling, enhances the entry of glucose carbons into the TCA cycle, promotes ATP production, and cell survival during detachment [48, 74]. Interestingly, the finding that overexpression active forms of AKT in PDK1-knockdown breast cancer cells are unable to rescue anchorage-independent growth indicates that PI3K could also be activated by PDK1, which is the downstream target of Ras/ERK signaling [107].

### Rho signaling pathway

Rho family small GTPases are key molecules regulating remodeling of the actin cytoskeleton. The central molecules in Rho signaling are RhoA, Rac1, and Cdc42, which can be activated by multiple signaling molecules, such as growth factor receptors, and mechanical forces [64, 108]. Active Cdc42 and Rac1 protect epithelial cells and fibroblasts from anoikis via activation of AKT and ERK signaling pathway [109, 110]. Rho-associated kinase (ROCK), the main effector in Rho signaling pathway, protects anchorage-independent survival, while ROCK inhibitor Y27632 reverses anoikis resistance [111, 112]. Y27632 also reduces entosis and protects cells from lysosomal cell death [28]. Furthermore, a report that ROCK inhibitors reduce damages and improve outcomes in retinal detachment, in vivo [113], also supports the critical role of Rho signaling pathway in anchorage-independent survival.

### Hippo signaling pathway

Hippo signaling plays a critical role in mechanical transduction in multiple cancers, for further information, see [114, 115]. YAP and TAZ, two key cotranscription factors in hippo signaling pathway, could be activated by αvβ3 integrin, while the activated YAP/TAZ transcriptionally upregulate GLUT3 expression and increase glucose uptake to support anchorage-independent survival of glioblastoma cells [116]. During detachment, cytoskeleton reorganization activates Lats1/2 and leads to YAP phosphorylation and inactivation, then induces anoikis in nontransformed cells [117]. Additionally, YAP could activate PI3K/AKT signaling pathway via transcriptional regulation of PI3Kcb, a catalytic subunit of PI3K/AKT signaling [118].

## The implication of anchorage-independent survival in cancer metastasis

Cancer metastasis is a process that cancer cells detach from the primary site, enter the vascular or lymphatic vessel, localize, and reproduce at remote sites [96, 119]. Thus, anchorage-independent survival is critical for the success of metastasis. While tumor heterogeneity endows tumor cells the potential to survive from various stresses, tumor cells successfully adapt to a stressed environment via activation of the above key signaling pathways that will take the priority of colonization and develop metastasis. Thus, therapies against the above processes or signaling pathways hold the potential to prevent or cure cancer metastasis (Table 1).

**Table 1:** The role of signaling pathways in anchorage-independent survival and their implication in the treatment of cancer metastasis.

Active signaling pathway	Functions during detachment	Activated in cancer metastasis	Efficacy of targeting inhibitors in metastatic patient or animal
Integrin/FAK/SFKs pathway	Protecting cells from anoikis and activating protective autophagy	Activated in a variety of metastasis, including breast cancer, lung cancer, melanoma, colorectal cancer, prostate cancer, glioblastoma, liver cancer [96]	Melanoma [176], breast cancer [177], colorectal cancer [178], ovarian cancer [179], hepatocellular carcinoma [180], non-small cell lung cancer [181], etc.
Ras/ERK signaling pathway	Attenuating cellular stress and enhancing anoikis resistance	Colorectal cancer [132, 135], germ-cell tumors [136], multiple myeloma [137], skin cancer [138, 139], melanoma [140] and lung cancer [141]	Thyroid cancer [142, 143], non-small cell lung cancer [144, 182], melanoma [145, 183, 184], colorectal cancer [185], biliary tract cancer [186]
PI3K/ATK signaling pathway	Enhancing nutrients uptake, decreasing ROS production and inhibiting anoikis	Colorectal cancer [135, 146], melanoma [147, 148], prostate cancer [149], lung cancer, breast cancer and renal cell carcinomas [150]	Endometrial cancer [155], breast cancer [156, 187–189], gastric cancer [190], cervical carcinoma [191], prostate cancer [192, 193], non-small cell lung cancer [194] and other cancers [195, 196]
Rho signaling pathway	Regulating cytoskeleton reorganization and mechanical transduction, protecting cells from anoikis and entosis	Lymphoma [157], colorectal cancer [158, 159], breast cancer [197, 198], liver cancer [199, 200], melanoma [201, 202], pancreatic cancer [203]	Nasopharyngeal cancer [160], pancreatic cancer [161], breast cancer [204], melanoma and colorectal cancer [162]
Hippo signaling pathway	Regulating mechanical transduction, enhancing nutrients uptake, protecting from anoikis and autophagy	Breast cancer [165, 205, 206], prostate cancer [117], lung cancer [207], colorectal cancer [208], melanoma [165], gastric cancer [163]	Breast cancer [165, 209] and melanoma [165]

### Integrin/FAK/SFK signaling in metastasis

Numerous studies prove that integrin/FAK/SFK signaling pathway plays a critical role in cancer metastasis [64, 96]. Hence, inhibition of integrin/FAK/SFK signaling seems prospective to prevent metastasis. However, integrin inhibitors failed to show monotherapy efficacy in patients with advanced or metastatic cancer in several clinical trials [120–124]. Given that, researchers explore the combination therapy with other drugs in different cancers and some report combinational efficacy and acceptable toxicity in advanced lung cancer [125–127]. Same as integrin inhibitors, limited clinical efficacy is documented in advanced cancer patients receiving a single FAK/SFK inhibitor [128–131]. Collectively, integrin/FAK/SFK targeting therapy still holds the prospect in metastatic cancer treatment but needs further investigation. It is important to keep in mind that there is more to integrin/FAK/SFK signaling inhibition therapy. First, most integrins play a redundant role in both adhesion and signaling transduction, and there is also compensatory upregulation of a nontargeted integrin. Both of which make it difficult to block these processes with a single drug. Moreover, it is extremely hard to achieve acceptable toxicity in metastasis treatment due to the critical function of integrins in normal tissue homeostasis.

### Ras/ERK signaling in metastasis

Genetic extinction of oncogenic Kras signaling results in specific elimination of invasive and metastatic disease while allowing for sustained primary tumor growth [132]. Moreover, oncogenic transformation of Ras in NIH/3T3 generates a metastasis phenotype [133, 134]. Recently, it is clear that Ras/ERK signaling is functionally required for cancer metastasis in colorectal cancer [132, 135], germ-cell tumors [136], multiple myeloma [137], skin cancer [138, 139], melanoma [140], and lung cancer [141]. Clinically, Ras/ERK signaling pathway inhibitors show antitumor activities in untreated BRAF mutant unresectable or metastatic cancers [142–145] and prolong the patients’ survival. Due to increasing application in primary or metastatic cancers, however, treatment resistance of Ras/ERK pathway inhibitors turns to be an inevitable and frustrating issue.

### PI3K/AKT signaling in metastasis

Genomic profiling reveals that metastasis specific genetic mutation or activation are enriched in PI3K/AKT signaling pathway in multiple types of cancer, including colorectal cancer [135, 146], melanoma [147, 148], prostate cancer [149], lung cancer, breast cancer, and renal cell carcinomas [150]. Moreover, kinases in PI3K/AKT signaling pathway are activated in circulating tumor cells (CTCs), which are derived from primary sites and developed several years before metastasis [151, 152]. Animal experiments report the efficacy of PI3K/AKT signaling inhibitors in reducing metastasis [153, 154]. Clinically, PI3K/AKT inhibitors demonstrate combinational therapeutic efficacy with other therapies in metastasis or advanced cancers [155, 156]. However, same as Ras/ERK pathway inhibitors, drug resistance is the major obstacle for PI3K/AKT signaling inhibitors in metastasis.

### Rho signaling in metastasis

Accumulating evidences indicate that increased activity or expression of Rac1, Cdc42, and ROCK enhances metastatic potential of cancer cells, in vitro and in vivo [157–159]. Several preclinical studies report the efficacy of Rho/ROCK inhibitors in treating metastatic nasopharyngeal, pancreatic carcinoma, and breast cancer [160, 161]. Furthermore, *Huang* et al. demonstrate that Fasudil, an FDA-approved RhoA/ROCK inhibitor, could reduce metastasis through facilitating the arrest of CTCs [162]. These results imply the potential of Rho signaling inhibitors in metastasis treatment.

### YAP/TAZ signaling in metastasis

An abundance of studies demonstrate the high activation of YAP/TAZ signaling pathway in metastatic tumor [115, 117]. In CTCs, YAP transcriptionally upregulates Rho GTPase activation protein 29 (ARHGAP29) and hence promotes metastasis of gastric cancer [163]. Moreover, YAP-dependent metabolic adaptation promotes lymph node metastasis in melanoma patients [164]. Currently, Verteporfin, a YAP/TAZ–TEAD interaction inhibitor, suppresses the prometastasis effect of YAP in breast cancer and melanoma [165]. However, the clinical application of verteporfin in cancer patients is restricted because of global toxicity and low solubility. Also, very slight penetration into the brain, which is one of the most common metastatic sites, is another challenge for verteporfin-treating metastasis.

### Targeting mechanical transduction and adaptation

During metastasis, cancer cells suffer from multiple mechanical forces, including fluid shear stress when circulating within the vessel systems and cell deformation when passaging through the microvasculature [62, 67, 166]. More than 90% of cancer cells died due to these mechanical forces [166, 167]. The range of fluid shear stress varies from vessels that are 0.64–12 dyn/cm^2^ in the lymphatic system, 4–30 dyn/cm^2^ in arteries, and 1–4 dyn/cm^2^ in veins [62]. It is well established that high mechanical force leads to cell cycle arrest and even death [62, 168]. In addition, cancer cells undergo mechanical transduction as reviewed above against such restraints [62, 168]. There are also mechanical adapted strategies against mechanical stresses. For example, membrane stretch induced by microvascular deformation induces PANX1 opening and activation, leading to increased ATP releasing, in turn, the released ATP supports cell viability by activating P2Y receptors in microvascular metastatic breast cancer [67]. Hence, inhibition of mechanical transduction and adaptation would decrease the burden of CTCs and hence prevent metastasis.

### Suppression of metabolism and antioxidant response

The expression of nutrient transporters such as lipid transporter and antioxidant defense-related genes, is increased in metastatic cancer cells, which promote colonization at lipid-rich tissue, in vivo [169]. Tasdogan and colleagues report that inhibition of MCT1, which transports lactate to maintain pentose phosphate pathway and redox balance, depletes CTCs in melanoma and reduces metastatic burden in patient-derived xenografts [170]. These results imply that inhibition of metabolism and antioxidant response poses the potential in treating cancer metastasis. However, controversary conclusions are found in antioxidant inhibition therapy. Some others report that both systemic antioxidant dosing and activation of cell-intrinsic antioxidant pathways promote metastasis in animal models of melanoma [171], breast cancer [172], and lung cancer [173, 174].

### Entosis-targeted therapy in metastatic cancer

It is well documented in various types of cancer that the presence of entosis in metastatic cancers is much common compared with primary cancers [28, 39, 175]. Given that entosis can generate distinct functional cellular entities by division or releasing from cell-in-cell structure, it is reasonable that entosis not only promotes cell viability during metastasis, but also contributes to its heterogeneity and malignancy after colonization. Entosis-targeting therapy presents a high possibility fortifying metastasis treatment. Except Rho/ROCK signaling pathway [28], however, the molecular mechanism of cell-in-cell structure formation and entotic cell death is largely unknown. Recently, Hayashi group reports that genetic features, including TP53 mutation, Kras amplification, and MYC amplification, are significantly associated with entosis in pancreatic cancer [39]. Without doubt, effective in vitro and in vivo models will boost the understanding of the role and regulation of entosis in metastasis, and help metastasis treatment.

## Conclusion

Detachment, the initial step of metastasis, is a stressed event and during which cells suffer from multiple stresses, including loss of growth stimuli, altered mechanical force, cytoskeletal reorganization, diminished nutrient uptake, and increased ROS production. Those failed to adapt to these stresses will undergo various forms of cell death, such as anoikis, autophagy, cell cycle arrest, and entosis. Consequently, the majority of cells die; however, a very small number of cells survive, in vitro and in vivo. The survived cells colonize and develop metastasis at the remote sites. Hence, detachment acts as selection and evolution power that imposes cancer cells metastatic potential and promotes malignancy during cancer development.

Previous studies demonstrate that a variety of signaling pathways are upregulated during detachment and required for anchorage-independent survival, such as integrin/FAK/SFKs, Ras/ERK, PI3K/AKT, Rho, YAP/TAZ, and other cooperative signaling pathways. Those pathways are also found to be highly activated in metastatic cancer samples. Thus, the mechanism study of cellular and genetic adaptation to anchorage-independent survival will shed light on the understanding of metastasis and implications for metastasis treatment. Indeed, therapies targeting metabolism, antioxidant response, mechanical transduction, and the related signaling pathways show impressive efficacy in various metastasis models. Clinically, some specific inhibitors against integrin/FAK/SFKs, Ras/ERK and PI3K/AKT signaling pathways could improve the outcome of patients with metastasis. However, apart from limited clinical efficacy and potential toxicity in metastasis treatment, treatment resistance turns to be a critical challenge and obstacle for cancer treatment. Therefore, mechanism and regulation study of anchorage-independent survival will help reveal the mechanism of drug resistance, explore the combinational efficacy, and improve metastasis patient’s survival. Worthy nothing, the efforts to explore the appropriate models, in vitro and in vivo, will accelerate and broaden our understanding of anchorage-independent survival and cancer metastasis.
